# Targeting Mitochondrial and Brain Injury Markers in Acquired Brain Injuries: A Randomized, Double-Blind, Placebo-Controlled Study with Melatonin

**DOI:** 10.34172/apb.2022.013

**Published:** 2021-04-03

**Authors:** Bahareh Hakiminia, Babak Alikiaie, Fariborz Khorvash, Sarah Mousavi

**Affiliations:** ^1^Department of Clinical Pharmacy and Pharmacy Practice, School of Pharmacy and Pharmaceutical Sciences, Isfahan University of Medical Sciences, Isfahan, Iran.; ^2^Department of Anesthesiology and Intensive Care, Alzahra hospital, Isfahan University of Medical Sciences, Isfahan, Iran.; ^3^Department of Neurology, Alzahra hospital, Isfahan University of Medical Sciences, Isfahan, Iran.

**Keywords:** Brain injury, Melatonin, Mitochondria, Oxidative stress

## Abstract

***Purpose:*** Oxidative stress-induced mitochondrial damage is the main event in acquired brain injuries (ABI). This study aimed to evaluate the effects of melatonin, a mitochondria-targeted antioxidant, on mitochondrial and brain injury markers, and the clinical outcomes of patients with ABI.

***Methods:*** In this randomized controlled trial, intensive care unit (ICU) or neurology patients with ABI (n=60) received melatonin (21 mg/day) or placebo tablets, within the first 72 hours of injury onset for five days. As a primary endpoint, serum levels of malondialdehyde (MDA), S100B and C-reactive protein (CRP) were compared at baseline, and after five days’ intervention. Secondary endpoints included assessment of Glasgow Coma Scale and Sequential Organ Failure Assessment (at the end of day 5), Rancho Los Amigos Revised Scale and modified Rankin Scale (at the end of month 3), the duration of mechanical ventilation, the lengths of ICU and hospital stays, and in-hospital and three-month mortality.

***Results:*** There were no significant effects of melatonin on the primary and secondary outcomes. However, the subgroup analysis showed a significant reduction in S100B in patients with non-traumatic brain injuries, receiving melatonin versus placebo (p: 0.016).

***Conclusion:*** This study showed that melatonin supplementation in the early phase of brain injury had no significant effects on the injury markers and clinical outcomes of patients with ABI. However, it reduced the level of S100B in the non-traumatic subgroup. Further larger-scale studies are needed to determine the effects of melatonin on the ABI and its subgroups.

## Introduction

Patients with acquired brain injuries (ABI) often suffer from a wide range of physical, cognitive, and psychological disabilities.^1^ Oxidative stress-induced neuronal damage is the main injury mechanism in different types of ABI.^2,3^ Following a brain injury, a wide spectrum of events occur, leading to the generation of large amounts of reactive oxygen species (ROS). There are several antioxidants in the brain, such as superoxide dismutase (SOD), catalase (CAT), and glutathione peroxidase (GPx), to combat oxidative stress.^4^ While the increased activity of the antioxidant defense system occurs after injury, an injured brain cannot neutralize the over production of free radicals, resulting in oxidative damage to neuronal lipids, proteins, and nucleic acids.^5^

Mitochondria, as important organelles for the neurological function, are significantly susceptible to free radical damage. Mitochondrial damage is the major cause of secondary injury, associated with traumatic brain injury (TBI)^5^ and is a decisive event after brain ischemia.^6^ Following the damage to the mitochondria, energy production is disturbed, which leads to the formation of ROS, overactivation of glutamate receptors, and the rapid influx of calcium ions into the mitochondria. The overloaded Ca^2+^ results in the opening of the mitochondrial permeability transition pores, changing the permeability and causing apoptotic cell death.^4,7^

Considering the critical role of oxidative stress in the molecular mechanisms of brain injury, researchers have introduced several agents with antioxidant properties as favorable candidates for the treatment of brain injuries. A group of agents, such as statins, N-acetyl-L-cysteine, curcumin, citicoline, growth factors, mannitol,^8^ vitamin C, and vitamin E,^9^ has been reported as neuroprotectants in experimental and clinical trials of stroke and TBI. However, there are numerous limitations in the administration of exogenous antioxidants, such as limited permeability through the blood-brain barrier (BBB), instability of these agents, the narrow therapeutic window, and toxicity at higher doses.^4^ Melatonin (N-acetyl-5-methoxytryptamine) is a lipophilic molecule, which can simply pass the BBB^10^ and enter intracellular organelles, such as mitochondria.^11^ Melatonin is safe and non-toxic, even at high doses,^10^ and is known as a neuroprotective agent with remarkable antioxidant properties. The antioxidant effects of this agent have been studied in experimental models of TBI,^12^ ischemic stroke,^13^ intracerebral hemorrhage (ICH),^14^ and subarachnoid hemorrhage (SAH).^15^ Also, its potential therapeutic effects have been explored in human studies of neonatal hypoxic-ischemic encephalopathy^16^ and ICH.^17^ It acts as a free radical scavenger and antioxidant, decreasing the ROS and reactive nitrogen species and increasing the levels of antioxidant enzymes.^18^ Moreover, it mitigates the level of malondialdehyde (MDA) and prevents oxidative-induced cellular damage during brain injuries.^13,19^ It also exerts positive effects on the mitochondrial function by improving the electron transport, especially by inhibiting the direct mitochondrial oxidative damage, thereby leading to the prevention of apoptosis.^18^

MDA, as the end product of lipid peroxidation, is the best marker of oxidative stress.^20^ The increased levels of MDA have been reported in acute ischemic stroke^21^ and TBI.^22^ S100B, as a known biomarker of brain injury, is associated with poor outcomes.^23^

Since the mitochondria are the main organelles in the occurrence of oxidative stress-induced brain damage in patients with ABI, it seems that administration of antioxidants targeting the mitochondria is of value in this population. While melatonin, a mitochondria-targeted antioxidant,^11^ has been reported as a potential neuroprotective agent in several experimental studies, human studies are scare in this area. Therefore, we conducted this double-blind, placebo-controlled, randomized trial to assess the possible effects of early melatonin administration on the levels of biomarkers, including MDA, S100B, and C-reactive protein (CRP) in patients with brain injuries.

## Materials and Methods

This study was designed as a double-blind, placebo-controlled, randomized study, which was performed during June 2019 to December 2019. The study protocol was approved by the Ethics Committee of Isfahan University of Medical Sciences (IUMS). The trial was registered at IRCT (Iranian Registry of Clinical Trials) with the code number of IRCT20081208001497N8. The informed consent was obtained from all patients. The participants were selected from those patients who were referred to AL-Zahra hospital of Isfahan University of Medical Sciences, Isfahan, Iran.

Patients were eligible for inclusion, if they met all of the following criteria: age ≥18 years old, diagnosed with TBI (skull fracture; brain laceration, contusion, or hematoma; SAH; ICH; intra-ventricular hemorrhage; or traumatic axonal injury) or non-TBI (due to strokes, infections, hypoxia, or brain tumors), identified within the first 72 hours of brain injury onset in the intensive care unit (ICU) or neurology ward, and proper function of the gastrointestinal tract (patients tolerated oral medications by gavage or mouth). Exclusion criteria were defined as follows: less than five days stay in the ICU or neurology ward, sensitivity reaction to the melatonin tablet, pregnancy, hepatic failure (class C according to Child-Pugh score), renal failure (need dialysis), severe heart failure (New York Heart Association (NYHA) classification III/IV), sepsis within first five days of admission or previous history of any types of brain injury.

### Random assignment

A research coordinator conducted the randomization and delivered the study drug. The participants and medical staff blinded to the treatment assignment. Eligible participants randomly assigned 1:1 to either the treatment group or the placebo group in accordance with the predefined randomization list with a block size of four.

### Treatment protocol

The treatment group received 21 mg (seven 3 mg tablets) of melatonin (Razak Co., Tehran, Iran) orally, as two divided daily doses (four tablets in the morning and three ones in the evening) for five continuous days. Patients in the control group received placebo (which was prepared in the pharmaceutical laboratory of the pharmacy faculty of IUMS) with the same dose for five continuous days. The placebo tablet was similar in size and color with the melatonin tablet. The investigator delivered drug or placebo in the same packaging containers. Investigator evaluated drug compliance by counting pills and patients with less than 80% compliance removed from the study.

### Outcome measures

The primary outcome was to evaluate the effects of melatonin on the injury biomarkers (MDA, S100B, and CRP) compared to the placebo group after five days’ intervention. Therefore, blood samples were attained from both groups’ participants (5 cc) at baseline before administration of drug or placebo and again after the last dose. Samples were centrifuged at 3000-4000 rpm for 10-15 minutes. After that, the serum was isolated and stored in a labeled microtube at -80°C. The serum MDA levels were determined by MDA Assay kit (Teb Pazhouhan Razi Co., Tehran, Iran) and the serum S100B levels were measured by Human S-100 Calcium Binding Protein B Assay Kit (Elabscience Biotechnology Co., Wuhan, China), using an enzyme-linked immunosorbent assay (ELISA) method in terms of the manufactures’ instructions. The values of CRP were obtained from hospital laboratory data.

Secondary outcomes included assessment of the duration of mechanical ventilation, the lengths of ICU and hospital stays, and in-hospital and three-month mortality, as well as the neurological, cognitive, and functional outcomes. In addition, sequential organ failure assessment (SOFA) score was calculated at the beginning and at the end of the intervention. The Glasgow Coma Scale (GCS) was compared at baseline and after five days’ intervention to assess the neurological state. The cognitive and functional states were measured by Rancho Los Amigos Revised Scale (RLAS-R) and modified Rankin Scale (mRS), respectively. These two scales were measured on the first day before the intervention based on hospital records and three months later through telephone interviews with patients or their relatives.

RLAS-R^24,25^ is a useful tool to provide a description of ten levels of cognitive and behavioral function in patients with brain injury as they recover from injury. It is simple and broadly accepted to use clinically. Scores 1 and 10 indicate “no response” and “Purposeful/ appropriate response”, respectively. This scale is used for categorizing recovery levels based on patient’s abilities to react to the stimuli and obey commands, as well as patient’s orientation, attention, memory, and communication traits.

As a functional outcome scale, mRS is a valuable tool for evaluating the degree of disability and dependence of those who suffer from brain damage. This seven-level scale with a score range of 0 to 6 is commonly used as an outcome measure in clinical trials. A score of 0 indicates “no symptoms” and a score of 6 indicates “death”. This evaluation tool is primarily used in stroke population,^26^ however, it has been also used in other types of ABI.^27^

A checklist consisting of all needed demographic and clinical data was filled out by a pharmacotherapy resident. In addition, cognitive and functional scores were determined under the supervision of a skilled nurse.

### Sample size calculation

According to the previous study,^28^ we expected melatonin to reduce the serum MDA level by the value of 7 µmol/L. We calculated the required sample size for an estimated dropout rate of 10%, a one-sided level of significance of α=5%, and a power of 80%, assuming the standard deviation (SD) of serum MDA as 5.5 µmol/L for both groups. A sample size of 20 patients in each group was estimated to be sufficient to detect a significant difference in serum levels of MDA and other biomarkers between both groups. To evaluate the differences in secondary clinical outcomes between both groups, a sample size of 30 patients in each group was considered.

### Statistical analysis

Statistical analysis was performed based on the intention to treat (ITT) principle. Continuous data were assessed for normality by the Shapiro-Wilk test. Normally distributed and non-normally distributed data are presented as the mean ± SD and median (first and third quartiles), respectively. Independent-samples *t*test and Paired-samples *t*-test were performed in order to compare normally distributed variables between and within groups, respectively. Mann-Whitney U test and Wilcoxon Signed-Ranks test were performed on non-normally distributed and ordinal variables for evaluating between- and within-group differences, respectively. Categorical variables are expressed as frequencies and percentages, and comparisons between groups were assessed by means of the Chi-square test or Fisher’s exact test, as appropriate. A value of *P* ≤0.05 considered statistically significant. All analyses performed using SPSS statistics software V24.0 (SPSS Inc; Chicago, IL, USA).

## Results and Discussion

**Figure 1:**
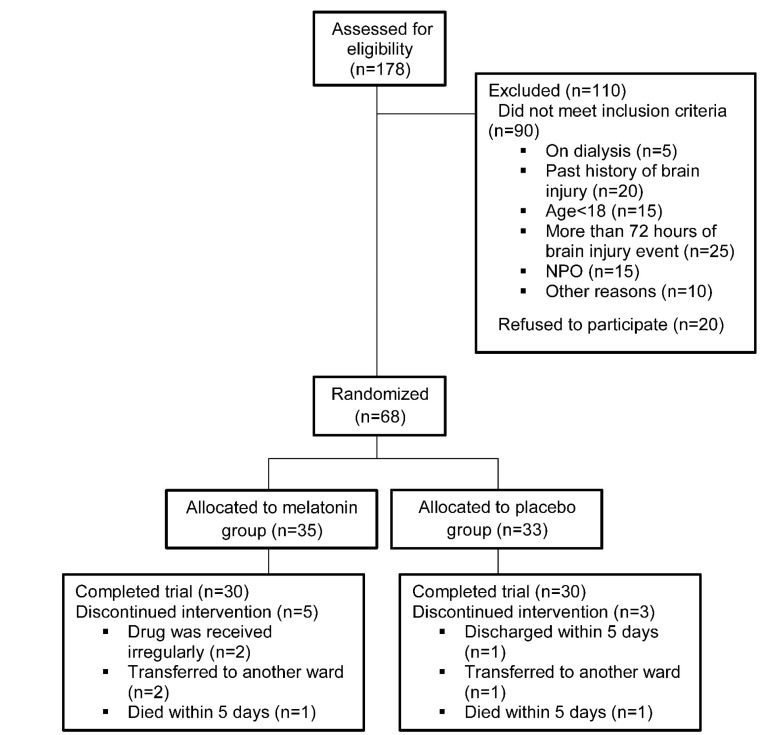
Progress through the stage of trial

Over the study period, of 178 brain-injured patients who were assessed for eligibility, 68 were randomly assigned (35 patients to melatonin and 33 patients to placebo group) with a ratio of 1:1. Thirty patients in each group completed the protocol. Reasons for interrupting the treatment are reported in Figure 1.

Table 1 shows the baseline demographic and clinical characteristics of the patients. As shown, the patients of two groups were matched in terms of all baseline values, including serum biomarkers and clinical scores.

**Table 1:** Baseline demographic and clinical characteristics of the study patients

**Characteristic**	**Melatonin** **(n=30)** ^#^	**Placebo** **(n=30)** ^#^	***P*** ** value** ^*^
Age (years)^a^	46.10±23.92 (18-97)	49.27±19.96 (18-93)	0.580
Male/female^b^	24/6	24/6	1.000
Admission's ward^c^
ICU	29 (96.67)	26 (86.67)	0.353
Neurology	1 (3.33)	4 (13.33)	
Diagnosis category^c^
Ischemic stroke	3 (10.00)	6 (20.00)	0.081^d^
Hemorrhagic stroke	5 (16.66)	11 (36.67)	
Brain tumor	2 (6.67)	0 (0)
Traumatic brain injury	20 (66.67)	13 (43.33)
Non-traumatic/traumatic^b^	10/20	17/13	0.119
Multiple trauma^c^	11 (36.67)	12 (40.00)	0.791
Laboratory data^e^
Serum creatinine (mg/dL)	1.04±0.32	1.02±0.22	0.889
BUN (mg/dL)	15.13±9.21	14.77±6.33	0.858
WBC (10^3^/mm^3^)	9.94±2.86	9.76 ±3.86	0.844
Hb (g/dL)	10.84±2.15	10.95±2.19	0.849
Platelet (10^3^/mm^3^)	119.23±43.46	126.79±43.45	0.507
AST (U/L)	47.25±43.07	32.00±14.59	0.252
ALT (U/L)	28.94±18.99	24.45±11.31	0.488
Albumin (g/dL)	3.29±0.49	3.35±0.58	0.690
CRP (mg/L)	80.28±20.74	76.28±33.68	0.882
GCS^f^	7 (5.75-10.5)	7.5 (6-15)	0.249
GCS ≤8^c^	21 (70.00)	17 (56.67)	0.422
GCS >8^c^	9 (30.00)	13 (43.33)	
APACHE II score^e^	14.96±5.43	12.46±6.17	0.116
SOFA^f^	6 (4-7)	5.5 (1.75-8)	0.419
RLAS-R^f^	3 (3-5)	4.5 (3-6)	0.073
mRS^f^	5 (5-5)	5 (5-5)	0.960
MDA (µmol/L)^f^	14.85 (10.82-24.82)	11.35 (9.35-17.84)	0.174
S100B (pg/mL)^f^	232.10 (74.18-458.54)	392.87 (123.35-481.68)	0.620

ICU: intensive care unit; BUN: blood urea nitrogen; WBC: white blood cell; Hb: hemoglobin; AST: aspartate aminotransferase; ALT: alanine aminotransferase; CRP: C-reactive protein; GCS: Glasgow coma scale; APACHE: acute physiology and chronic health evaluation; SOFA: sequential organ failure assessment; RLAS-R: Rancho Los Amigos revised scale; mRS: modified Rankin scale; MDA: malondialdehyde. ^#^The values of CRP, MDA, and S100B are referred to 20 patients in each group. APACHE II and SOFA scores were measured only in ICU-admitted patients; ^*^Independent-samples *t*-test and Mann-Whitney U test were used to compare parametric and nonparametric values, respectively. Chi-square test was performed to compare nominal values; ^a^Mean ± standard deviation (range); ^b^Number; ^c^Number (%); ^d^There were no significant differences regarding tumor types and their numbers between the two groups; ^e^Mean ± standard deviation; ^f^Median (first and third quartiles).

Table 2 shows the effects of interventions on the evaluating biomarkers after five days in the study subjects. As seen, melatonin was not significantly effective in reducing the levels of MDA, S100B, and CRP compared to the placebo.

**Table 2:** The effects of interventions on the measured biomarkers in the study patients

**Variable**	**Melatonin** **(n=20)**	**Placebo** **(n=20)**	***P*** ** value** ^*^	**Mean of difference (95% CI)**	**Effect size**
MDA (µmol/L)					
Baseline	19.67±12.98 14.85 (10.82-24.82)	14.29±7.82 11.35 (9.35-17.84)	0.457	2.75 (-4.65, 10.14)	0.239
End	15.94±8.62 14.45 (10.13-17.52)	13.31±8.92 11.26 (8.87-17.00)			
*P* value^**^	0.204	0.627			
S100B (pg/mL)					
Baseline	312.79±308.21 232.10 (74.18-458.54)	318.84±218.58 392.87 (123.35-481.68)	0.163	104.33 (-44.09, 252.75)	0.452
End	236.71±227.20 147.38 (60.50-406.28)	347.08±215.57 433.69 (167.96-527.29)			
*P* value^**^	0.204	0.526			
CRP (mg/L)					
Baseline	80.28±20.74 81.5 (69.50-94.25)	76.28±33.68 86 (65.25-89.25)	0.554	-8.07 ( -35.74, 19.60)	-0.229
End	75.28±31.96 74.5 (45.50-106.25)	63.21±28.68 75 (36.75-81.75)			
*P* value^***^	0.546	0.247			

CI: confidence interval; MDA: malondialdehyde; CRP: C-reactive protein. The values are presented as mean ± standard deviation and median (first and third quartiles); ^*^Independent-samples *t*-test (between-group comparison); ^**^Wilcoxon Signed-Ranks test (within-group comparison); ^***^Paired-samples *t*-test (within-group comparison).

Table 3 shows the effects of interventions on the clinical scores; GCS and SOFA (at the end of day 5), and RLAS-R and mRS (at the end of month 3). As shown, melatonin significantly improved scores compared to the baseline (*P*< 0.001). However, these effects were not statistically significant compared to the placebo group.

**Table 3:** The effects of interventions on the clinical scores in the study patients

**variable**	**Melatonin** **(n=30)** ^#^	**Placebo** **(n=30)** ^#^	***P*** ** value** ^*^
RLAS-R score
Before intervention	3 (3-5)	4.5 (3-6)	0.929
Month 3	10 (6.75-10)	10 (7.25-10)	
*P* value^**^	<0.001	<0.001	
mRS score
Before intervention	5 (5-5)	5 (5-5)	0.137
Month 3	3 (1-5)	3 (0-4)	
*P* value^**^	<0.001	<0.001	
GCS score
Before intervention	7 (5.75-10.50)	7.5 (6-15)	0.422
After intervention	9 (6-14.25)	12 (6.75-15)	
*P* value^**^	<0.001	0.015	
SOFA score
Before intervention	6 (4-7)	5.5 (1.75-8)	0.462
After intervention	4 (1-6)	3.5 (1-5.2)	
*P* value^**^	<0.001	<0.001	

RLAS-R: Rancho Los Amigos revised scale; mRS: modified Rankin scale; GCS: Glasgow coma scale; SOFA: sequential organ failure assessment.

The values are presented as median (first and third quartiles);^#^No level is assigned to the dead patients regarding RLAS-R score, so they were not included in the analysis of RLAS-R score at month 3. At month 3, RLAS-R score is referred to 22 and 28 patients in melatonin and placebo groups, respectively. At month 3, mRS score is referred to 28 and 29 patients in melatonin and placebo groups, respectively. SOFA score was measured only in ICU-admitted patients; ^*^Mann-Whitney U test (between-group comparison); ^**^Wilcoxon Signed-Ranks test (within-group comparison).

There was no difference in the duration of mechanical ventilation (*P*: 0.645) between study groups. Although the lengths of ICU and hospital stays were shorter in patients who received melatonin in comparison with the placebo group, they were not statistically significant (*P*: 0.987 and *p*: 0.719, respectively). Neither during hospitalization nor after three months, mortality rate was not significantly different between study groups (*P*: 0.492 and *P*: 0.313, respectively) (Table 4).

**Table 4:** The effects of interventions on the length of mechanical ventilation, lengths of ICU and hospital stays, and mortality in the study patients

**Outcome**	**Melatonin** **(n=30)** ^#^	**Placebo** **(n=30)** ^#^	***P*** ** value** ^*^
Duration of mechanical ventilation (day)^a^	3 (0.50-8)	3 (0-10)	0.645
Length of ICU stay (day)^a^	13 (9-22)	14 (6.75-27.25)	0.987
Length of hospital stay (day)^a^	16 (14-30)	19 (10.75-33.25)	0.719
Mortality rate during hospitalization, dead/alive^b^	2/28 (6.66)	0/30 (0)	0.492
Mortality rate after three months, dead/alive^b^	6/23 (20.69)	2/27 (6.89)	0.313

ICU: intensive care unit. ^#^At month 3, data regarding the life status of two patients (one in each group) could not be reached; ^*^Mann-Whitney U test and chi-square test were used to compare numerical and nominal values, respectively;^a^Median (first and third quartiles); ^b^Number (%).

The subgroup analysis showed no significant effect of the brain injury severity (GCS > or ≤8) on the effects of melatonin supplementation (results are not shown). In subgroup analysis based on the type of brain injury (traumatic or non-traumatic), no significant effects of melatonin on the primary and secondary outcomes occurred except for S100B levels. In patients with non-traumatic brain injuries the level of S100B was significantly reduced in the melatonin group as compared to the placebo group (*P*: 0.016) (Table 5). No adverse effects were reported from patients of melatonin and placebo groups.

**Table 5:** The effects of interventions on the level of S100B (pg/mL) based on the subgroup (traumatic/non-traumatic) analysis in the study patients

**Variable**	**Traumatic**		**Non-traumatic**	
	**Melatonin** **(n=14)**	**Placebo** **(n=10)**	**Melatonin** **(n=6)**	**Placebo** **(n=10)**
S100B (pg/mL)	Before intervention	215.09±217.91 116.57 (322.25)	366.20±232.06 458.54 (412.05)	540.77±385.24 544.45 (704.94)	271.47±204.95 222.35 (381.03)
	After intervention	211.60±154.55 147.38 (238.91)	330.51±286.65 424.91 (545.09)	295.28±358.02 144.10 (715.65)	363.65±123.79 433.69 (232.38)
*P* value^*^	0.706	0.016
Mean of difference (95% CI)	-32.20 (-207.10, 142.69)	337.66 (72.57, 602.76)
Effect size	-0.159	1.36

CI: confidence interval. The values of S100B are presented as mean ± standard deviation and median (interquartile range);^*^Independent-samples*t*test (between-group comparison).

This double-blind, placebo-controlled, randomized study showed that in adults with ABI, melatonin at a dose of 21 mg/day, administered within the first 72 hours of injury onset for five days, had no significant effects on the injury markers (MDA, S100B, and CRP), or the length of ventilation and hospitalization. Also, no significant effects were observed on the neurological, cognitive, and functional outcomes. However, the subgroup analysis showed a significant reduction in S100B in patients with non-traumatic brain injuries, receiving melatonin versus placebo; this finding is consistent with the result reported in a recent clinical trial on non-traumatic ICH patients.^29^

Previous studies have revealed the dysregulated secretion of melatonin in critically ill and TBI patients,^17,30^ besides the reduced level of melatonin in non-traumatic ICH patients.^31^ Although the exact cause of these impairments is not clear, several mechanisms have been suggested, such as the pineal gland dysfunction or consumption of melatonin as an endogenous antioxidant for neuroprotection.^31^ Moreover, it has been shown that the disease severity and the drugs used in the ICU affect melatonin secretion and decrease the plasma levels of melatonin.^32^

Oxidative stress-induced neuronal mitochondrial damage is the main injury mechanism in different types of ABI.^33^ Melatonin shows remarkable antioxidant properties through direct scavenging of free radicals and inducing the up regulation of antioxidant enzymes.^18^ Moreover, this mitochondria-targeted molecule^11^ has positive effects on the mitochondrial function by improving the electron transport, and especially by inhibiting the direct mitochondrial oxidative damage, thereby leading to the prevention of apoptosis.^18^ To the best of our knowledge, there is only one clinical study on the effects of high doses of melatonin on brain-injured patients. In this double-blind, randomized clinical trial by Dianatkhah et al,^17,29^ a total of 40 ventilated adult patients with non-traumatic ICH received 30 mg of melatonin or placebo within 24 hours of hemorrhage onset for five nights. According to the results, melatonin significantly reduced the level of S100B, the length of ICU stay, and the GCS score. Although the effects of melatonin on the duration of mechanical ventilation and the mortality rate were favorable, they were not significant; therefore, some of their results are similar to our study, while some are inconsistent. The discrepancy between these studies may be due to the use of a lower dose of melatonin in our study than in the mentioned study. Generally, it is assumed that a dose of melatonin higher than that needed for regulating the sleep-wake cycle can affect the mitochondrial function.^18,34^ It has been reported that higher doses of melatonin cause non-receptor-mediated effects, such as free radical scavenging activities and enhancement of the mitochondrial function at the supra-physiological levels.^35^ However, the exact dose is not established yet. In the present study, the insignificantly shorter duration of ICU and hospital stays was reported in the melatonin group. Moreover, an insignificant reduction in S100B was detected in the melatonin group, compared to the baseline; and a significant decrease was found in the serum levels of S100B in the non-traumatic subgroup of the melatonin group, compared to the placebo. Therefore, higher doses of melatonin may significantly affect the variables in these patients.

The time window for the administration of neuroprotective agents is a factor that should be considered. A secondary brain injury has a rapid and progressive course, particularly in the first few days^36^; therefore, timing of melatonin administration is important regarding the free radical formation. In a previous study, the therapeutic window for neuroprotection was reported up to four days following a stroke-induced brain injury.^37^ In our study, melatonin was administered up to 72 hours after the injury, whereas in the mentioned study, it was used earlier within 24 hours of injury, which might be the cause of its higher efficacy. Moreover, it should be mentioned that in our study, the patients received melatonin in two separate doses, that is, one dose in the morning and another at night; this is unlike the mentioned study that administered the total dose at night. When melatonin is administered in a single dose at night, its high dose may be more effective in replacing the reduced level of endogenous melatonin, considering the physiological secretion pattern.

It should be also noted that in these two studies, the subjects were not highly similar, which is another possible reason for the inconsistent results. We recruited patients with traumatic and non-traumatic brain injuries, while non-traumatic ICH patients were examined in the study by Dianatkhah and colleagues.

On the other hand, consistent with our results, another clinical trial showed that melatonin had no positive effects on all outcomes. Hosseinjani et al^38^ evaluated the potential effects of a high dose of melatonin on the outcomes of patients with sepsis, where mitochondrial dysfunction and oxidative stress are important aspects of the injury mechanism. They found that melatonin at a dose of 51 mg for five nights could not significantly reduce the S100B and CRP levels in ICU-admitted patients with sepsis, which is similar to our findings. Contrary to our results, they observed a significant improvement in the clinical scores (GCS and SOFA) of patients with sepsis, who received melatonin compared to control group. Variations in the study populations (ABI vs. sepsis), the dose of melatonin, and the frequency and time of administration may be responsible for the inconsistent results.

Melatonin has been reported as a potential neuroprotective agent in the treatment of neurodegenerative diseases.^39^ The protective effects of this agent against oxidative stress, induced by reductions in the ROS and MDA levels and maintenance of antioxidant enzymes, have been reported in different animal studies of brain injuries. In this regard, Kerman et al^19^ examined the neuroprotective effects of melatonin at a total dose of 10 mg/kg in rabbits with head trauma-induced oxidative stress. Melatonin was injected intraperitoneally in four divided doses 20 minutes before and during trauma and one and two hours later. At 24 hours after the brain trauma, higher antioxidant enzymes (CAT and GPx) and lower MDA levels were reported in melatonin-treated animals as compared to the control group. In another animal study, 5 mg/kg of intraperitoneally-injected melatonin reduced the level of MDA in the ischemic brain regions of treated rats, compared to the placebo group at 60 minutes following the middle cerebral artery occlusion.^13^ Melatonin also protected the brain and other organs as a free radical neutralizer in clinical conditions, where mitochondrial dysfunction, oxidative stress, and lipid peroxidation were present.^40^ It has been also shown that melatonin is effective in reducing the serum MDA levels in newborns with sepsis.^41^ In contrast to these studies, although a higher reduction of MDA was observed in the intervention group compared to the placebo group, the difference was not significant. It seems that differences in the study population and condition (human, adult, and brain injury vs. animal, neonate, and sepsis), duration of intervention, and the route of administration may be the cause of discrepancy between the results.

As mentioned earlier, in the current study, melatonin could not significantly reduce the level of S100B after the intervention compared to the placebo group, whereas the subgroup analysis showed a significant reduction in S100B in patients with non-traumatic brain injuries, which is consistent with the results reported by Dianatkhah et al.^29^ The antioxidant properties of melatonin were proposed as one of the possible mechanisms of its effectiveness. Overall, S100B is mostly produced by astrocytes in the central nervous system (CNS).^42^ Some studies reported that the presence of multiple trauma might be problematic when S100B is evaluated as a brain injury marker.^43^ Although S100B can be released into the serum from other organs after injury, extracerebral S100B has a more rapid clearance than the CNS-released S100B.^42^ It has been suggested that the initial large amount of S100B, released from the extracranial tissue into the serum, undergoes a rapid clearance within the first hours after injury; whereas, the CNS secretion is lengthier^42,44^; therefore, the CNS-originated S100B remains elevated for days. Also, the contributory role of multiple trauma in the release of S100B was normalized within 12 hours.^42^ It seems that multiple trauma cannot be a confounding factor in the interpretation of melatonin effects on this biomarker in this study. It is worth mentioning that in our study, the number of patients with multiple trauma was nearly equal in both study groups.

Mechanical ventilation is a crucial part of treatment for a considerable number of patients, admitted to the ICU.^45^ Sleep disorder, as a major issue in ICU-admitted patients, has some deleterious effects, such as difficulty weaning from mechanical ventilation, prolonged duration of ICU stay, and increased in-ICU mortality.^32^ Reductions in melatonin secretion and plasma levels were observed during mechanical ventilation, associated with difficult weaning.^46,47^ Sedating and analgesic agents, routinely prescribed for the management of patients, potentially prolong mechanical ventilation, whereas melatonin offers sedative, hypnotic, and analgesic effects, without any negative effects on the respiratory function. Administration of melatonin was also associated with the reduced concomitant use of sedative agents, resulting in fewer side effects and shorter mechanical ventilation.^48-50^ Furthermore, one of the complications of mechanical ventilation, especially at high volume or pressure, is ventilator-associated lung injury, probably due to the formation of free radicals.^51^ The protective and antioxidant effects of melatonin were found in an animal study of acute lung injury.^52^ Moreover, recently, ramelteon, as an agonist of melatonin receptors, was found to reduce the serum levels of MDA and inflammatory cytokines and exerted protective effects in an animal model of ventilator-induced lung injury.^51^ Overall, melatonin may have potential effects on weaning from mechanical ventilation. However, in this study, melatonin supplementation in an early phase of brain injury had no significant effects on the duration of mechanical ventilation, the length of ICU or hospital stay, and the mortality rate (in-hospital and three-month mortality rates). It should be noted that in this study, the sleep quality and dose of sedative and analgesic agents were not measured or compared between the study groups, which could affect the effectiveness of melatonin.

Acquired injuries of the brain have frequent negative effects on cognitive and motor functions, as well as emotional and behavioral expressions, all affecting the patient’s recovery and quality of life.^53^ Other outcomes investigated in the present study for the first time, were three-month cognitive and functional disabilities, assessed by the RLAS-R and the mRS, respectively. These scales have been used as outcome measure scales in different brain injury studies to evaluate the effects of interventions.^27,53^ In the current study, there were no significant desirable changes in the melatonin group as compared to the placebo group regarding the three-month outcomes, although RLAS-R and mRS indices showed significant positive changes compared to the baseline.

Adequate absorption, plasma level, and safety are other important issues that should be considered in exogenous supplementation with high doses of melatonin in critically ill, brain-injured patients. Previous studies have evaluated the pharmacokinetic aspects of oral supplementation with melatonin in critically ill patients. In one study which was conducted to evaluate the effects of exogenous oral administration of melatonin on the escalation of total antioxidant capacity in critically ill patients, despite the early phase of the disease, adequate enteral absorption was reported.^30^ In another study, Rouini et al^31^ suggested that the oral administration of melatonin could correct the reduced level of melatonin in ICH patients. They conducted a single-blind randomized clinical trial, in which 24 critically ill adult patients with non-traumatic ICH were equally divided into treatment and control groups. The treatment group received 30 mg of melatonin within 24 hours of hemorrhage onset for five days. Decreased levels of baseline melatonin were reported in both groups, compared to the healthy subjects. Only the melatonin group reached the corrected melatonin plasma concentration on the fifth day of the study. This study also indicated that oral melatonin at a dose of 30 mg is safe in humans, with good absorption; the peak concentration time (T_max_) was about 45 minutes, even in the acute phase after brain injury. Overall, melatonin was well-tolerated and reported as a safe supplement, even at high doses in several other human studies.^10,54^ Expectedly, no adverse effects of melatonin supplementation were observed in our study.

The main limitations of this study were its small sample size, the short duration of the intervention, and probably the inadequate dose of melatonin to affect the parameters significantly. Moreover, this study was a single-center research, and also other factors that might affect the outcomes, such as the presence or absence of rehabilitation were not considered. On the other hand, the strength of this study was its double-blind, randomized, placebo-controlled design.

Overall, in the current study, melatonin was initiated at a daily dose of 21 mg within 72 hours of insult and continued for five consecutive days in patients with ABI. To the best of our knowledge, this is the first study evaluating the effects of high supra-physiological doses of oral melatonin on the biomarkers of mitochondrial and brain injuries and the three-month cognitive and functional outcomes in patients with ABI.

## Conclusion

Mitochondrial damage due to oxidative stress is a major event, associated with secondary injury in ABI; on the other hand, administration of antioxidants may affect this process. The present results showed that short-term daily supplementation with 21 mg of melatonin, as a mitochondria-targeted antioxidant administered within 72 hours of injury onset, could not affect the injury biomarkers, the length of ventilation and hospitalization or clinical scores of patients with ABI. However, it reduced the level of S100B in the non-traumatic subgroup. Further studies using higher doses of melatonin, a larger sample size, and longer durations of the intervention and follow-up are needed to determine the effects of melatonin on the ABIs and its subgroups.

## Ethical Issues

This study was in accordance with the ethical standards of the responsible committee on human experimentation and with the Helsinki Declaration of 1975, as revised in 2008.The study was approved by the Isfahan University of Medical Sciences’ ethics board, and patients’ data were kept confidential.

## Conflict of Interest

None to be declared.

## Acknowledgments

This study was granted by the research deputy of Isfahan University of Medical Sciences (grant number: 397732).
